# Single-cell sperm transcriptomes and variants from fathers of children with and without autism spectrum disorder

**DOI:** 10.1038/s41525-020-0117-4

**Published:** 2020-02-21

**Authors:** Delia Tomoiaga, Vanessa Aguiar-Pulido, Shristi Shrestha, Paul Feinstein, Shawn E. Levy, Christopher E. Mason, Jeffrey A. Rosenfeld

**Affiliations:** 1 Department of Physiology and Biophysics, Weill Cornell Medicine, New York, NY USA; 2 The Feil Family Brain and Mind Research Institute, Weill Cornell Medicine, New York, NY USA; 3 Hudson Alpha Institute for Biotechnology, Huntsville, AL USA; 4 Hunter College, City University of New York, New York, NY USA; 5 The HRH Prince Alwaleed Bin Talal Bin Abdulaziz Alsaud Institute for Computational Biomedicine, Weill Cornell Medicine, New York, NY USA; 6 The WorldQuant Initiative for Quantitative Prediction, Weill Cornell Medicine, New York, NY USA; 7 Rutgers Cancer Institute of New Jersey, New Brunswick, NJ USA; 8 Department of Pathology, Robert Wood Johnson Medical School, New Brunswick, NJ USA

**Keywords:** Molecular medicine, Predictive markers

## Abstract

The human sperm is one of the smallest cells in the body, but also one of the most important, as it serves as the entire paternal genetic contribution to a child. Investigating RNA and mutations in sperm is especially relevant for diseases such as autism spectrum disorders (ASD), which have been correlated with advanced paternal age. Historically, studies have focused on the assessment of bulk sperm, wherein millions of individual sperm are present and only high-frequency variants can be detected. Using 10× Chromium single-cell sequencing technology, we assessed the transcriptome from >65,000 single spermatozoa across six sperm donors (scSperm-RNA-seq), including two who fathered multiple children with ASD and four fathers of neurotypical children. Using RNA-seq methods for differential expression and variant analysis, we found clusters of sperm mutations in each donor that are indicative of the sperm being produced by different stem cell pools. Finally, we have shown that genetic variations can be found in single sperm.

## Introduction

Single-cell RNA sequencing (scRNA-seq) allows for the discovery and investigation of many cellular subtypes.^1^ To date, this technique has not been employed on human germline tissues such as ova or mature sperm (spermatozoa). Spermatozoa are a challenging cell type for the investigation of RNA at the single-cell level, as they differ from typical somatic cells in several aspects. First, they are transcriptionally restricted cells, retaining only a small quantity of RNA per cell (~50 fg) which exists in a fragmented or partially degraded state.^2–4^ Second, transcription ceases during the spermatid stage of spermiogenesis and sequential displacement of histones by transition proteins and eventually protamines (PRM1 and PRM2) takes place, along with nuclear remodeling.^5^ Third, spermatozoa exhibit a compact nucleus, minimal cytoplasm, a head-housed acrosome, and a mitochondria-heavy midpiece, plus a long tail of ~50 µm. This particular cell morphology, paired with the ability to move rapidly, can also prove challenging for capturing of single sperm, especially with microfluidic devices. These features taken as a whole make sequencing of single-spermatozoa RNA more challenging, but also create an ideal paradigm for investigating transcriptome composition of sperm at a mature stage where new RNAs are not being produced and the ones retained may be of functional importance to the oocyte.

The functions of the majority of RNAs in sperm remain unknown.^6^ However, there has been evidence that spermatozoa may have a role in the regulation of early embryonic development by delivering functional RNAs to the oocyte during fertilization.^7–9^ During the final stage of spermatogenesis (spermiogenesis), chromatin remodeling takes place, leading the nucleosome from a histone-bound to a protamine-bound configuration, involving histone-variants replacement of histones, hyperacetylation, transient DNA breaks and repair, transition proteins (TNPs) replacing histones and protamines PRM1 and PRM2 replacing TNPs. Prm1 and Prm2 are, by far, the most abundant transcripts we (and others) found in expressed mature sperm. Due to their abundance relative to other sperm RNAs, they have been studied extensively in bulk assays. Departures from typical ratios of PRM1/PRM2 (protein), Prm1/Prm2 (mRNA), protamine/histones, and protamine protein/mRNA in sperm have been shown to correlate with altered reproduction-related phenotypes.^10,11^ Furthermore, retained sperm histones associate with telomeric sequences and are the first sperm structures to respond to oocyte signals,^12^ and histone marks are heavily implicated in fertilization and development.^13^

Using the 10× Genomics Chromium platform, we performed single-cell RNA sequencing of six donor sperm samples obtained from a sperm bank with approval for research use (see “Methods”). This platform has been extensively validated across a wide range of sample types, and has been shown to be extremely consistent when the same sample is analyzed multiple times.^14,15^ As a test case for our single-cell sperm sequencing, we decided to sequence sperm from donors who have had children with autism and those who have had only had children without the disorder. ASD is a complex neurodevelopmental condition with an often undetermined complex etiology^16^ and is classified as a paternal age effect (PAE) disorder, since increased paternal age is associated with higher ASD risk.^17^ Typically, two to three new mutations arise in sperm germ cells with each year of the father’s age.^18,19^ Although hundreds of mutations increasing the risk of autism have been identified, they only account for <20% of known causes, with many cases having an unknown component, limiting diagnostic breadth.^20^ Here, we used a unique approach in performing single-nucleotide variant (SNV) calling on RNA-seq data at single-cell resolution to uncover variants in the sperm of donors. Our data represent the first proof of principle for single-cell RNA sequencing and mutation detection at single-cell resolution in spermatozoa, which set the stage for identifying patterns of paternal transmission risk of the ASD phenotype. Finally, by enabling both expression mapping and allele-specific variant calling, it also shows the potential for an RNA-seq-based biomarker test in sperm and for potential use as a diagnostic or investigational tool of other neurodevelopmental and PAE-transmitted phenotypes.

## Results

### Sequencing results and filtering parameters

**Fig. 1:**
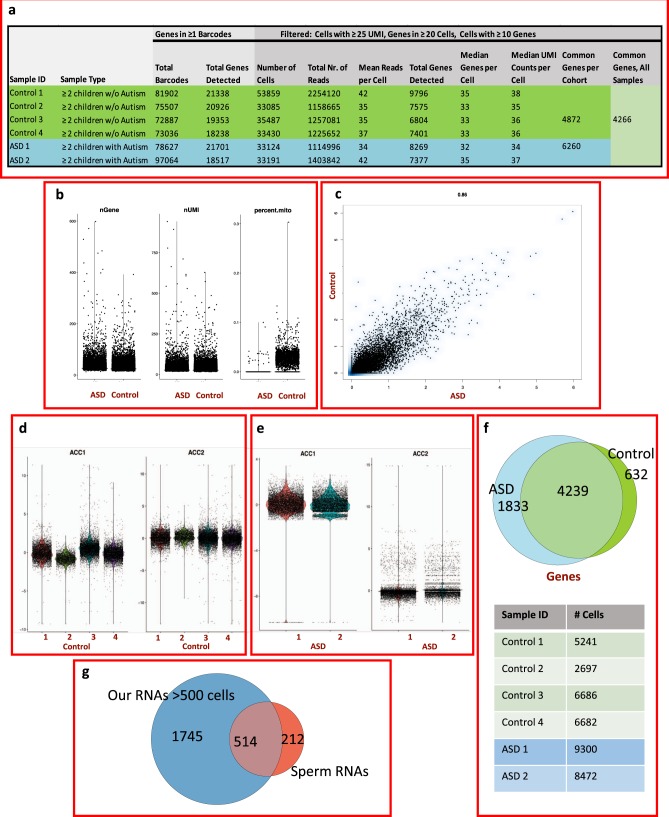
Single-sperm RNA-seq profiles and metrics. **a** Metrics for (i) barcodes containing one or more genes and (ii) for the filtered set used in downstream analysis that includes all cells with 10 or more genes, all genes present in at least 20 cells, and all cells with at least 25 unique molecular identifiers (UMI). **b** The distribution of genes and unique molecular identifiers and percent mitochondrial genes/cell in the groups in the filtered set used for analysis. **c** Cell scatter plot comparing the range in scaled average expression in each cohort and the correlation statistic for the whole set. Each feature represents a gene expression value averaged across all single cells in the group. **d**, **e** Violin plots of the cohort-specific features in the data sets post-alignment of the data sets. **f** The number of common and unique genes in each group in the post-alignment set. **g** A Venn diagram of the number of genes expressed in our single-cell sperm sequencing as compared with bulk sperm sequencing.

Among the six sperm samples, the number of barcodes with at least one gene varied between 72,507 in sample Control 3 and 97,064 in sample ASD2, while the number of genes detected varied between 18,238 in Control 4 and 21,701 in sample ASD1 (Fig. 1a). For the downstream analysis, we excluded cells under the 25 UMI per cell threshold, cells with fewer than 10 detected genes, and genes detected in <20 cells and cells with mitochondrial genes exceeding 40% of the total, based on the typical mature sperm mitochondrial content.^5^ We detected 4872 common genes among the four Control samples and 6260 common genes between the two ASD samples (Fig. 1a). Across all samples in the filtered data set passing the thresholds, 4266 genes are common (Fig. 1a). The distribution of genes, the number of unique molecular identifiers (nUMI), and percent mitochondria reads per cell in the filtered set are shown in Fig. 1b. Multiple sample alignment and normalization for the integration of the data sets were performed with *Seurat* R package v.2.1.0 and 2.2.0,^21^ leading to correlated expression values per cell across groups (Fig. 1c) and comparable post-alignment distributions for features common among both groups (Fig. 1d, e). After alignment, the number of cells in each sample were reduced (Fig. 1f), and also the number of genes to 1833, 4239, and 632 in the ASD unique, shared, and control feature groups.

To determine whether our results were a consequence of using a single-cell assay, we compared them to gene expression data from bulk sperm samples. We took the set of genes that were expressed in at least 500 cells in our samples (2259 genes) and compared them to genes that were reported to be expressed in published RNA-seq data of three mature sperm samples.^4^ There was a significant overlap of 514 genes that were found to be expressed in both data sets, but also genes that are expressed only in one set (Fig. 1g; Supplementary Data 2).

### Transcript abundances and DEG analysis

The most abundant transcripts present in the largest number of cells in both control and ASD samples include: PRM1, PRM2, TSSK6, DNAJC4, NUPR2, CRISP2, and SMCP (Table 1; Supplementary Data 3). Protamines PRM1 and PRM2 replace ~85% of the histones in human sperm during maturation, at the spermatid stage^22^ and, as expected, are the highest-expressed transcripts in our spermatozoa samples, appearing both in the largest number of cells and at the top of the list of the gene-specific average expression values in our samples. These abundant transcripts are highly specific to sperm, and have lower or no expression in Human Universal Reference (UHUR) controls.^4,23^

**Fig. 2:**
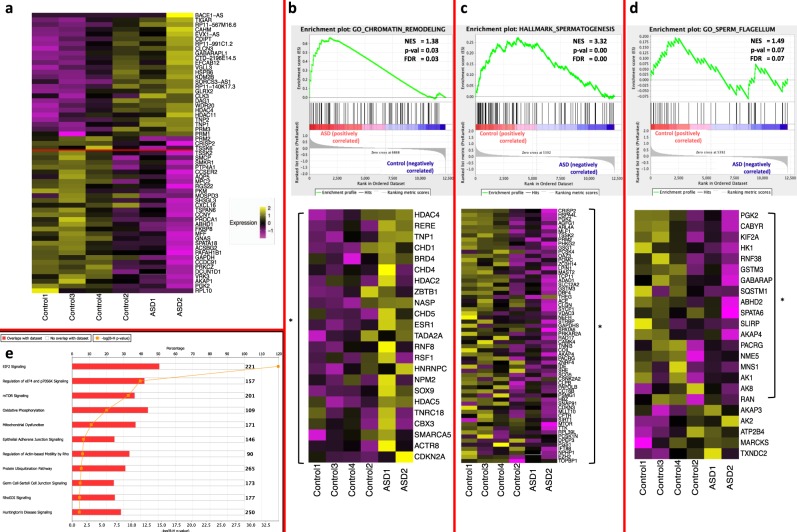
Distinctive profiles between groups. **a** Heatmap of representative DEGs (aligned set) from the top and bottom of the list ranked by average log fold change, between the ASD and the Control samples. Each rectangle represents the scaled average expression of the single cells for a specific gene. **b**–**d** GSEA enrichment plots showing: (**b**) Chromatin remodeling (**c**) Spermatogenesis (**d**) Flagellum. (**e**) Canonical pathways enrichment analysis in Qiagen IPA, the red bar represents the ratio of the # of DEGs in the pathway to total genes in the pathway.

Overall, after normalization, the average number of molecules expressed per cell between the two groups was highly correlated (*R*^2^ = 0.86) (Fig. 1c), indicating overall high correlation between their transcriptomes. Differential expression analysis comparing the cells in the ASD and the Control samples in the aligned set revealed 2114 differentially expressed genes (DEGs, *q*-value < 0.05, Bonferroni-adjusted *p*-value for multiple hypothesis testing), of which 1247 were increased and 867 were decreased in the ASD samples. When the minimum threshold for marker expression was set such that a transcript was present in at least 1% of cells in either group, the list was reduced to 688 differentially expressed genes between the ASD and the Control groups, with 345 genes showing an increase and 343 genes showing a decrease in the ASD set. (Fig. 2a; Supplementary Data 4).

To perform a broader, exploratory analysis of the two groups of donors, we also calculated the differentially expressed genes for the entire Seurat gene set (Wilcoxon rank-sum test, see “Methods”), finding 1000 DEGs to have a significant *q*-value < 0.05 (Supplementary Data 5) and performed Gene Set Enrichment Analysis,^24^ uncovering enriched gene sets and pathways in Control vs. ASD samples, with representative results shown in Fig. 2b–d. Notably, we uncovered enrichments in epigenetic regulation, such as chromatin remodeling, H3K4me3 and H3K27me3, and histone deacetylation activity, as well as neurodevelopmental and sperm-related processes, such as sperm flagellum, spermatogenesis, male gamete generation, and mitochondria functions. The extended GSEA test results are shown in Supplementary Data 6.

### SNV analysis

**Fig. 3:**
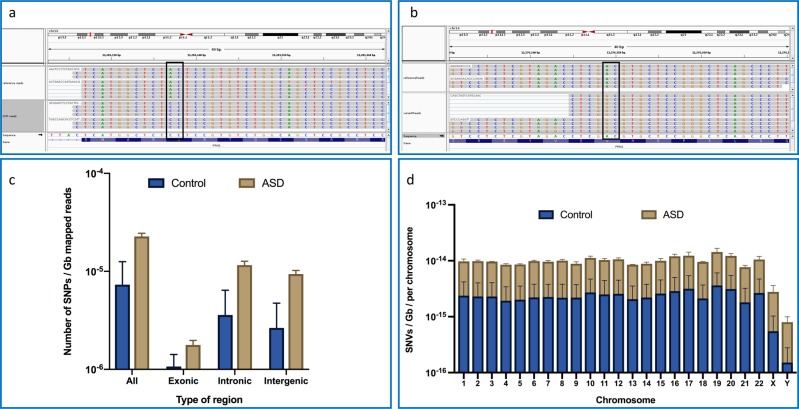
SNVs from single-sperm cells. Using the Integrative Genome Viewer (IGV), data are shown from alignments to the reference genome from single cells. **a** PRM1 variants are shown as coverage tracks (rows) with the reference genome on top and the donor variants on the bottom row. **b** Same as **a**, but shown for the same donor in PRM2. **c** The number of SNVs found in exonic, intronic, intergenic, and all three regions for the bulk RNA-seq analysis, normalized to the total number of reads mapped to the transcriptome by the size of the read. Values are shown in a log10 scale. **d** The number of SNVs found in the bulk RNA-seq analysis with an allele frequency < 0.001 in gnomAD per chromosome, normalized to the total number of reads mapped to the transcriptome by the size of the read and to the chromosome length. Values are shown in a log10 scale.

To explore the mutational landscapes of the individual sperm cells, we developed a new method for calling variants in scSperm-RNA-seq data (see “Methods”). First, we examined the distribution of variations across the cells. For each sample, there were between 194 and 302 SNPs present, with non-synonymous SNPs found in genes including CARHSP1, CRISP2, DNAJC4, NUPR2, PRM1, PRM2, and SMCP. To increase the stringency and to remove false positives, we further filtered the data to only include variants that were found in at least 100 sperm cells. At this level, we only found non-synonymous SNV in the PRM1 and PRM2 genes (Fig. 3a, b). Notably, we were able to discern distinct mutations in the sperm from individual cells. To our knowledge, this is the first detection of such variants from single-cell sperm RNA.

Given recent reports of using 10× Genomics single-cell RNA-seq data for calling variants within genes and nearby regions,^25^ we next examined the overall distribution of variants in the ASD and control samples. For this purpose, we carried out variant calling at bulk level (see “Methods”), obtaining a list of variants present in each genomic region. We first compared the total number of called variants per region between the ASD and control groups, which seemed not only to be higher overall but also in exonic, intronic, and intergenic regions (Fig. 3c). Despite the small sample size for this type of analysis, the *p*-value for each comparison was close to significance (*p*-value = 0.053). Furthermore, when comparing the number of rare variants, defined as those absent or present with an allele frequency <0.001 in the Genome Aggregation Database (gnomAD),^26^ this trend still holds true across both autosomes and sex chromosomes (Fig. 3d).

## Discussion

These results demonstrate that scSperm-RNA-seq is a promising method to profile gene expression and mutational dynamics of the transcriptomes of individual sperm cells. As expected, the highly expressed genes in both the ASD and Control samples displayed an overlap with genes in pathways for sperm maturation, DNA binding, early embryonic development, cell growth, and proliferation. Although the DEG and pathway differences observed between the cohorts can provide potential leads or serve as biomarkers for sperm health or function, they will need to be validated in a larger donor cohort size for a proper interpretation.

Nonetheless, the differences between the small cohorts reveal distinct expression landscapes and pathways, such as enrichment of genes from IPA canonical pathway analysis for mTOR signaling and eIF2 regulation in the ASD samples (Fig. 2e). The mTOR signaling pathway has recently been highlighted as a potential target of autism,^27^ while the eIF2 pathway is involved in the inhibition of CREB, a transcription factor required for long-lasting synaptic plasticity and long-term memory, and has recently been investigated in connection with several neurodegenerative disorders.^28^

Importantly, given sufficient depth from sequencing, which is now routine in terms of NGS platforms, we have shown that an investigation of sequence variants in RNA from sperm is possible. We were able to identify these features by sequencing sperm at single-cell level and by mapping individual gene expression across thousands of single cells and clusters of cells. Notably, rare mutational differences of single sperm would be not discovered from bulk RNA sequencing of the sperm. For example, performing differential expression statistical testing at the single-cell level, across thousands of cells simultaneously allows for the identification of differentially expressed features that may have a relatively small fold change across groups taken as a whole, but that are strong drivers in a subpopulation of cells.

These results serve as a proof of principle that single-cell sequencing can be done on likely any cell in the human body, even sperm. In order to determine whether there is truly a genetic marker in sperm for ASD, a much larger set of samples will need to be assessed in order to provide statistical power. In addition, since there are strong indicators that the markers may be found in SNVs rather than in gene expression differences, there might be value in doing single-cell sperm DNA sequencing in addition to single-cell sperm RNA sequencing.

## Methods

### Samples

Sperm samples were purchased from a private sperm bank in the USA. A summary of the donor’s ethnicity and year of birth are listed in Supplementary Data 1. Since the donor sperm samples at the sperm bank are purchased by women to use for insemination, many of the donors have a large number of children. Interestingly, there are cases where a donor will have one of his many children having ASD with the rest being unaffected, indicating that there are other causes of autism besides paternal genetics. For this reason, we required that our ASD donors have evidence of at least two children with ASD. The control donors needed to have multiple children with no evidence of ASD. The samples were of IUI quality, purified by the sperm bank with the standard protocol for enrichment of mature, motile spermatozoa, and were shipped on liquid nitrogen from the sperm bank to the lab where they were thawed for analysis. All of the donors signed an informed consent with the sperm bank agreeing to the inclusion of their samples in a biobank and that those samples could be used for research without the need for further consent. This research was approved by the New England IRB. We have complied with all relevant ethical regulations in this research for samples from a biobank.

### Cell preparation

Frozen sperm cell vials were obtained from the sperm bank. These samples were prepared according to the standard procedures of the sperm bank to remove somatic cells and to improve sperm quality. These techniques include swim-up and density gradient centrifugation.^29,30^ Sperm cells were provided with expected post-thaw cell count. Frozen cells were rapidly thawed in a 37 °C water bath. Thawed cells were centrifuged at 300 rcf for 10 min and washed twice with 1× PBS containing 0.04% bovine serum albumin, then resuspended in PBS at room temperature.

### Single-cell library construction and sequencing

Cell suspension post-washing was loaded on the 10× Chromium System (10× Genomics, Pleasanton, CA) for single-cell isolation into Gel Bead Emulsions (GEMs) as per the manufacturer’s instruction in Chromium Single Cell 3′ Reagent Kits v2 User Guide, Rev A^14^ using Chromium Single Cell 3′ Solution (Chromium Single Cell 3′ Chip Kit v2 [PN-120236], Gel Bead kit v2 [PN-120235]. The input cells per channel in the chip were targeted around ~1 million cells, based on provided initial post-thaw cell count from the sperm bank. However, the loss of cells during washing would reduce the actual cell counts to input significantly less than targeted cells.

Sperm samples that successfully generated proper GEMS were further processed for GEM-RT incubation, cDNA amplification and subsequent single-cell library construction using Chromium™ Single Cell 3′ library Kit v2 [PN-120234] following the manufacturer’s protocol. Barcoded final libraries were quantified by Qubit® 2.0 Fluorometer (Invitrogen) and qPCR (KAPA Biosystems Library Quantification kit), and fragment size profiles were assessed from Agilent 2100 BioAnalyzer. All libraries were sequenced on Illumina Hiseq 2500 with 2 × 100 paired-end kits using following read length: 26 bp Read 1, 8 bp i7 Index, and 98 bp Read 2.

Cellranger (v 1.2) single-cell pipeline (https://support.10xgenomics.com/single-cell-gene-expression/software/overview/welcome) was used for demultiplexing libraries, using cellranger mkfastq to generate FASTQ files. STAR alignment, barcode/UMI processing, and counting were conducted by the Cellranger count pipeline. Barcode, UMI, and duplicate sorting are further described.^14^

### Data analysis

For the data analysis, we excluded cells under the 25 UMI/cell threshold, cells with fewer than 10 detected genes, and genes detected in <20 cells. Cells with mitochondrial genes comprising >40% were excluded. Multiple pairwise alignment to remove batch effects and allow for integrated analysis was performed on all samples for each cohort as described and implemented in R toolkit Seurat (Seurat v.2.1.0 and 2.2.0*)*.^21^ Briefly, the filtered ASD and Control data sets were randomly subsampled to 12,000 cells per sample, including 24,000 cells total for ASD and 48,000 cells for the Control data set. The cells were subsampled based on common genes, the expression normalized, scaled and aligned into a conserved low-dimensional space using nonlinear warping algorithms. Canonical correlation vectors are aligned to normalize for differences in feature scale, an approach robust to shifts in population density. Variable genes were detected across the data sets. The ASD data set includes two samples, ASD1 with 9300 cells and ASD2 with 8472 cells, while the control data set contains four samples, Controls 1–4, each with 5241 cells, 2697 cells, 6686 cells, and 6682 cells, respectively.

We performed differentially expressed gene (DEG) analysis between all the single cells in the aligned data set as well as between all the cells in the filtered nonaligned data set (Bonferroni-adjusted *p*-values). For detecting differentially expressed genes between the sperm samples from fathers of children with ASD and those of children without ASD, we performed a Wilcoxon rank-sum test at single-cell level. A t-distributed stochastic neighbor embedding (t-SNE) analysis to visualize cells in a two-dimensional space was done. Based on the relative position of the cells on the t-SNE plot, unsupervised graph-based clustering was performed with the *FindClusters* function in Seurat, and unique cluster marker genes were identified. Further, we also calculated differentially expressed genes on the filtered set, without performing canonical correlation analysis (CCA)-based alignment between the samples, as described above. This gene set was used for running gene set enrichment analysis, in pre-ranked mode and with standard parameters and MSigDB sets.^24^

### Variant calling

The BAM file produced by the CellRanger software is not designed to easily allow for variant calling, and needs to be modified. In particular, the reads from each individual cell are marked with the CB tag in the BAM file, whereas a typical BAM file would record each set of reads from the same source as a read-group with the RG tag. We developed a cleaning algorithm (convertBAMfull.perl) that converted the BAM file and also removed any of the cells that had <100 reads per cell. We then used the Freebayes^31^ variant caller on the converted BAM files, and we removed any variants with a quality of <20 and required that a SNP be present in at least ten individual single cells. Thus, our SNPs were relatively rare in the sperm population, but were not only found in an individual cell.

The code for variant pruning is at: https://github.com/jeffr100/singleCellSperm.

### Variant calling, annotation, and analysis for bulk RNA-seq

Genetic variants were called using the Broad Institute’s GATK^32^ Best Practices for bulk RNA-seq variant calling. Duplicates were marked, and aligned reads were sorted using Picard tools. The SplitNCigarReads was used to split reads into exon segments and to clip reads overhanging intron regions. Variants were called using the HaplotypeCaller, and single-nucleotide polymorphisms (SNPs) were extracted using SelectVariants. Hard filtering was carried out using VariantFiltration to remove artifacts due to clusters of at least three SNPs in windows of 35 bp, as recommended by the Broad Institute. Finally, variants with a coverage < 20X for the alternative to the reference genome were not included in the analysis. The remaining variants were annotated using the most updated version (96) of Ensembl Variant Effect Predictor (VEP).^33^

Downstream analyses were performed using the processed data as input. First, the total number of variants was calculated per region (i.e., exonic, intronic, intergenic and all three) based on the annotations provided by VEP. For each sample, all variant counts were normalized to the reads mapped to the transcriptome according to the following formula:

$${\mathrm{Normalized}}\,{\mathrm{number}}\,{\mathrm{of}}\,{\mathrm{variants}}_{{\mathrm{sample}}\_i} = \frac{{{\mathrm{Number}}\,{\mathrm{of}}\,{\mathrm{variants}}_{{\mathrm{sample}}\_i}}}{{{\mathrm{Reads}}\,{\mathrm{confidently}}\,{\mathrm{mapped}}\,{\mathrm{to}}\,{\mathrm{the}}\,{\mathrm{transcriptome}}_{{\mathrm{sample}}\_i} \times {\mathrm{read}}\,{\mathrm{size}}}}$$

VEP filter was then used to extract rare variants for each sample, that is, those variants absent or present in the Genome Aggregation Database (gnomAD)^26^ with allele frequency < 0.001. Next, the total number of rare variants per chromosome was calculated and normalized following the subsequent formula:

$${\mathrm{Normalized}}\,{\mathrm{number}}\,{\mathrm{of}}\,{\mathrm{rare}}\,{\mathrm{variants}}_{{\mathrm{sample}}_i,{\mathrm{chr}}_j} = \frac{{{\mathrm{Number}}\,{\mathrm{of}}\,{\mathrm{rare}}\,{\mathrm{variants}}_{{\mathrm{sample}}_i}}}{{{\mathrm{Reads}}\,{\mathrm{confidently}}\,{\mathrm{mapped}}\,{\mathrm{to}}\,{\mathrm{the}}\,{\mathrm{transcriptome}}_{{\mathrm{sample}}_i} \times {\mathrm{read}}\,{\mathrm{size/length}}\,{\mathrm{of}}\,{\mathrm{chr}}_j}}$$

Mean and standard deviation were calculated for the ASD and control groups, and one-sided Wilcoxon rank-sum test was used to determine statistical significance between groups under the hypothesis that the number of SNVs is greater in ASD samples.

### Reporting summary

Further information on research design is available in the Nature Research Reporting Summary linked to this article.

## Supplementary information

Supplementary Data Captions

Supplementary Data 1

Supplementary Data 2

Supplementary Data 3

Supplementary Data 4

Supplementary Data 5

Supplementary Data 6

Reporting Summary Checklist

## Data Availability

The sequencing data for this study have been uploaded to the EGA archive with ID: EGAS00001004035.

## Code availability

All code used for this paper has been cited in the Methods section. The code for variant pruning is available at https://github.com/jeffr100/singleCellSperm, and is freely released and completely open-access without any retained license.
