# Supplementary material for: Single-cell sperm transcriptomes and variants from fathers of children with and without autism spectrum disorder
Source: NPJ Genom Med. 2020 Feb 21;5:14. doi: 10.1038/s41525-020-0117-4 (PMC7035312; doi:10.1038/s41525-020-0117-4)
Supplement: Supplementary file 1 — Supplementary Data Captions [file 41525_2020_117_MOESM1_ESM.pdf]

Supplementary Data 1. Year of birth and ethnicity of the donors

Supplementary Data 2. Genes expressed in single cell sperm and bulk sperm

Supplementary Data 3. Top transcripts expressed in ASD and control samples

Supplementary Data 4. Genes differentially expressed between ASD and control samples

Supplementary Data 5. 1,000 DEGs to have a significant q-value < 0.05

Supplementary Data 6. Extended GSEA test results
